# Morbillivirus-associated unusual mortality event in South Australian bottlenose dolphins is largest reported for the Southern Hemisphere

**DOI:** 10.1098/rsos.160838

**Published:** 2016-12-21

**Authors:** C. M. Kemper, I. Tomo, J. Bingham, S. S. Bastianello, J. Wang, S. E. Gibbs, L. Woolford, C. Dickason, D. Kelly

**Affiliations:** 1 South Australian Museum, North Terrace, Adelaide, South Australia 5000, Australia; 2 Australian Animal Health Laboratory, Commonwealth Scientific and Industrial Research Organisation, Private Bag 24, 5 Portarlington Road, Geelong, Victoria 3220, Australia; 3 Gribbles Veterinary Laboratory, 33 Flemington Street, Glenside, South Australia 5065, Australia; 4 Lot 30A, Seaview Road, Yatala Vale, South Australia 5126, Australia; 5 Animal and Veterinary Sciences, University of Adelaide, Roseworthy, South Australia 5371, Australia; 6 Biosecurity SA, Primary Industries and Regions South Australia, Research Centre, Lenswood, South Australia 5240, Australia; 7 Department of Environment, Water and Natural Resources, GPO Box 1047, Adelaide, South Australia 5001, Australia

**Keywords:** unusual mortality event, cetacean morbillivirus, Australia, Indo-Pacific bottlenose dolphin, environmental perturbation

## Abstract

Cases of morbillivirus have been recorded in the Southern Hemisphere but have not been linked to significant marine mammal mortality. Post-mortems were conducted on 58 carcasses (44 Indo-Pacific bottlenose dolphins, two common bottlenose dolphins, 12 short-beaked common dolphins) from South Australia during 2005–2013, including an unusual mortality event (UME) in St Vincent Gulf Bioregion (SVG) during 2013. Diagnostic pathology, circumstance of death, body condition, age and stomach contents were documented for Indo-Pacific bottlenose dolphins. At least 50 dolphins died during the UME, 41 were Indo-Pacific bottlenose dolphins and most were young. The UME lasted about seven months and had two peaks, the first being the largest. Effect on the population is unknown. Diagnostic testing for morbillivirus was conducted on 57 carcasses, with evidence for infection in all species during 2011–2013. All tested UME bottlenose dolphins were positive for cetacean morbillivirus (CeMV), and the pathology included interstitial pneumonia, lymphoid depletion and syncytia. Concurrent pathologies, including lung parasite and fungal infections, and severe cutaneous bruising were observed in many dolphins. The event coincided with elevated water temperatures, a diatom bloom and significant fish die-offs. We conclude that the cause for the UME was multifactorial and that CeMV was a major contributor.

## 1. Introduction

An unusual mortality event (UME) of marine mammals is defined in the United States Marine Mammal Act as ‘a stranding that is unexpected; involves a significant die-off of any marine mammal population; and demands immediate response’ [1]. These events have been documented in cetaceans, pinnipeds and sirenians [2,3] and can be multispecies in nature. UMEs caused by biotoxins appear to be increasing during the last 40 years, although changing mammal abundance and inconsistent effort to report and investigate all events may make it more difficult to document true frequency and causal factors [4]. The first documented UME dates to the early 1900s and most have occurred since about 1980 [2,5]. Some events have been linked to biotoxins produced by harmful algal blooms [5,6] and others to morbillivirus [7] and naval activities [8], but for many the cause was not determined [2]. In 2009, two UMEs were documented in Australia: a small event involving Indo-Pacific bottlenose dolphins, *Tursiops aduncus*, linked to dolphin morbillivirus (DMV) and presumed poxvirus infections in the Swan River, Western Australia [9,10], and an abnormally high number of humpback whale, *Megaptera novaeangliae*, mortalities along the Western Australian coast [11]. A third, suspected, UME was observed in Western Australian Indo-Pacific bottlenose dolphins during 2008–2009 [12].

In a review of emerging infectious diseases of cetaceans worldwide, Van Bressem *et al.* [13] discussed four categories of pathogens: viruses (including cetacean morbillivirus (CeMV), papillomavirus and poxvirus), *Brucella* spp., *Toxoplasma* spp. and *Lacazia loboi* (lobomycosis). Pathogens that infect cetaceans may be host-specific (e.g. viruses) or host-opportunistic (e.g. bacteria, fungi and protozoa). Van Bressem *et al.* [13] concluded that environmental degradation may have disturbed the evolutionary equilibrium between cetaceans and their pathogens, thus making some populations more susceptible to disease and mortality. The causes of UMEs are likely to be complex and involve a broad spectrum of factors in synergy. Van Bressem *et al.* [13] noted that there is mounting circumstantial evidence that chemical pollution has increased the frequency and severity of some diseases in cetaceans and pinnipeds. Evidence for morbillivirus has been found in cetaceans from all ocean basins but sizable mortality events have not been documented for the Southern Hemisphere [3,5].

Morbillivirus has been identified in six cetacean species from Australian waters [3,14]. Van Bressem *et al*. [7] reported that one of five common bottlenose dolphins, *Tursiops truncatus*, collected in Tasmania during 1997 tested positive for DMV-specific antibodies. Stone *et al*. [14] identified antibodies to CeMV in four species of delphinid and one baleen whale, all from southern Queensland and northern New South Wales and dating back to 1985. Stone *et al*. [15] also reported a fatal case of a common bottlenose dolphin from the same region. Viral genome and antigen of CeMV have been identified in Indo-Pacific bottlenose dolphins collected during the Swan River UME in Western Australia during 2009 [10].

Toxoplasmosis has been identified in two Australian cetaceans: the Australian humpback dolphin, *Sousa sahulensis* (*Sousa chinensis* in Bowater *et al*. [16]) and Indo-Pacific bottlenose dolphins [17]. At present, it is not considered a significant cause of cetacean mortality in Australia. However, there are no targeted surveys to identify its presence, therefore, its true prevalence and impact is not known.

To detect a UME, it is essential to have consistent, long-term monitoring of mortalities [4] so that variations from the usual pattern can be identified. In Australia, stranding networks are usually run by state and territory wildlife agencies but there is rarely a comprehensive post-mortem programme of mortalities. In South Australia (SA) a stranding network began in 1990 and is coordinated by South Australian Museum (SAM), with input from other government and non-government agencies. Trends in stranding patterns have been summarized by Segawa & Kemper [18] and circumstance of death (including a review of diseases) by Kemper *et al*. [19]. Post-mortems have been conducted since the beginning of the stranding programme, with the most detailed results from 2005.

The aim of this study was to document an unprecedented dolphin mortality event that occurred in SA during 2013, and to identify patterns and possible causes associated with the event. The study included some data for mortality of SA dolphins prior to 2013. The duration and geographical extent of the UME, the species and ages of the animals involved, and their stomach contents are presented. Results of the diagnostic investigation to determine causes of death are described. A brief description of the epidemiology of morbillivirus during the event is also presented. In this study, we focus on the biology and causes of the UME; details of the pathology findings of the cases will be presented in a separate publication.

## 2. Material and methods

The taxonomy adopted in this paper follows Kemper *et al*. [20] in which the species name applied to the inshore bottlenose dolphin is *T. aduncus*, not the Burrunan dolphin, *Tursiops australis*, as has been suggested by Charlton-Robb *et al*. [21]. The latter is not currently recognized as a valid species [22]. The term bottlenose dolphin refers to either both species or to carcasses identified only to genus level, i.e. *Tursiops*.

### 2.1. Study area

SVG and Spencer Gulf/North Spencer Gulf (SG/NSG) are large, shallow inverse estuaries characterized by limited water circulation, high salinity and high water temperature [23]. They include three of eight biogeographical regions in SA [24]. SVG Bioregion includes Investigator Strait and Backstairs Passage, both having more oceanic influences than the Gulf *sensu stricto*.

The cetacean fauna of SA includes oceanic and coastal species [20]. The Indo-Pacific bottlenose dolphin and short-beaked common dolphin, *Delphinus delphis*, are resident in SVG [25] and SG [26]. Indo-Pacific bottlenose dolphins occur along the coast to the east and west of the gulfs, where they are broadly sympatric with the common bottlenose dolphin. Since the common bottlenose dolphin is rarely recorded in SVG [25], we have assumed that dolphins identified as the bottlenose dolphin, *Tursiops* sp. (i.e. identified using only photos) in this study were the Indo-Pacific bottlenose dolphin.

### 2.2. Stranding records

In this paper, the term ‘stranding’ refers to both live-stranded dolphins and carcasses of dead animals. Dolphin strandings from around SA were recorded between 1990 and 2014. Table 1 summarizes data for 2013, when 72 events were recorded. A post-mortem examination was performed on 45 carcasses (three species). To identify when morbillivirus first appeared in SA, 13 additional dolphin carcasses (two species) were selected, on the basis of histopathological findings (e.g. interstitial pneumonia), from archived samples from dolphins collected in SVG and elsewhere in SA during 2005–2012. Summary data are presented for all post-mortemed carcasses that were included in the study (electronic supplementary material). For dolphins that were collected, species identification was confirmed by examining skull and postcranial features. If not examined by post-mortem, species, sex and age were assigned using photographs and descriptions provided by observers. In these cases, limited information was gained on pathology (e.g. skin lesions, haemorrhaging) and overall body condition.

**Table 1.** Number of dolphin strandings recorded and collected from SA during 2013. (Numbers in parentheses are animals collected and are included in the total.)

	Indo-Pacific bottlenose dolphin	common bottlenose dolphin	bottlenose dolphin	short-beaked common dolphin	unidentified dolphin	total
SVG	37 (31)	0	4 (0)	3 (3)	6 (0)	50 (34)
outside SVG	7 (4)	2 (2)	1 (0)	7 (5)	5 (0)	22 (11)
total	44 (35)	2 (2)	5 (0)	10 (8)	11 (0)	72 (45)

### 2.3. Post-mortem procedures and gross pathology

Standard post-mortem procedures were performed [27]. Total body length (tip of upper jaw to notch in the tail fluke) and body weight were recorded. Reproductive status was assessed by gross and/or histological examination of ovaries, uteri, mammary glands, testes and epididymides.

Body condition was assessed during post-mortem, and/or by examining photographs of whole animals. Four features were noted: presence/absence of a dorsal concavity behind the head; presence/absence of a concavity along the dorso-lateral surface of the body, indicating reduced muscle mass; presence of convexities where the transverse processes of caudal vertebrae appear along the peduncle; and clear indications of ribs in the thoracic region. Body condition indices were then assigned: robust = no features observed; slightly emaciated = 1–2 features; very emaciated = 3–4 features; CBD = cannot be determined owing to advanced decomposition or missing body parts.

Representative tissue samples (lung, lymph nodes, spleen, brain, thymus, liver, kidney, heart, skin, pancreas, adrenal, stomach, intestine, testis, mammary gland) from the major organ systems, as well as detected lesions, were collected and preserved in a variety of ways: 10% neutral buffered formalin, frozen, 70% ethanol.

Internal parasites were preserved in 10% formalin and/or 70% ethanol prior to species identification at the SAM. Carcasses that arrived unfrozen during 2013 were sampled for bacterial and fungal infections by swabbing the blowhole, rectum, mouth, eye socket and/or surface lesions (*n* = 21) prior to post-mortem. In addition, fresh samples of lung and/or liver and/or kidney lesions were collected from nine dolphins. These were cultured for aerobic bacteria, fungi and mycobacteria, and later examined by Gribbles Pathology Ltd.

Carcass decomposition was rated according to the system developed by Geraci & Lounsbury [27], which describes gross external and internal signs of autolysis. For carcasses necropsied in this study, 46 were assigned to code 2 (i.e. very fresh), 10 to code 3 and 2 to code 4 (substantial decomposition).

A cause/circumstance of death [19] was assigned to the 58 post-mortemed carcasses (electronic supplementary material) by integrating data on circumstances recorded at the time of collection and gross pathology. Criteria used for each category were as follows: known entanglement (removed from finfish aquaculture cage, purse seine net or monofilament shark net); probable entanglement (features consistent with death by entanglement e.g. net marks, food in oesophagus, severe and deep haemorrhage, large amount of fluid in body cavities, broken bones, dislocated spine) [28]; intentional killing (shotgun but did not die immediately); infectious disease (e.g. pneumonia, lymphadenitis, encephalitis); non-infectious disease (e.g. nephrosis); disease (combination of infectious and non-infectious, undiagnosable pathologies); unknown (e.g. no post-mortem, too decomposed, incomplete specimen, data inconclusive). Major cause of death (electronic supplementary material) was determined based on gross examination and histopathology without reference to immunohistochemistry (IHC) or polymerase chain reaction (PCR) results [29].

### 2.4. Histopathology and immunohistochemistry

Tissue samples were fixed in formalin, processed, sectioned and stained with haematoxylin and eosin (H&E), as per routine methods, then examined microscopically for histopathological lesions.

Sections of lung, spleen and lymph node, and occasionally other tissues such as thymus, kidney, heart, intestine, skin, adrenal and brain were tested by IHC for morbillivirus as described elsewhere [15]. The primary antibody used for IHC was a mouse monoclonal antibody directed against canine distemper virus nucleoprotein (CDV-NP,VMRD, Pullman, USA).

### 2.5. Polymerase chain reaction and sequencing analysis

Fresh-frozen and paraffin-embedded tissue samples were used for RNA extraction using MagMAX Viral RNA Isolation Kit (Thremo Fisher Scientific). Reverse transcriptase polymerase chain reaction (RT-PCR) for morbillivirus was conducted with extracted RNA as described previously [10,30] using the SuperScript III One-Step-RT-PCR System (Invitrogen). The positive PCR amplicons were subsequently sequenced by using the ABI BigDye Direct Sequencing method in Applied Biosystems 3500 xL Genetic Analyser (Applied Biosystems, USA).

### 2.6. Age

A relative age category was assigned to all collected carcasses, as well as some not collected, following Kemper & Gibbs [31]. Refinements were made to this system, i.e. ‘neonates’ were up to about three months of age and with neonatal folds; and length at weaning was 1.5 m for bottlenose dolphins and 1.3 m for short-beaked common dolphins. Dolphins that were recorded during 1990–2012 but not collected were assigned to approximate relative age categories: dolphins < 1.6 m = neonates, calves and juveniles, and dolphins  > 1.6 m = subadults and adults.

Age was estimated for 32 Indo-Pacific bottlenose dolphins collected from SVG during 2013 using incremental layers in the dentine of decalcified, thin-sectioned and stained teeth [32]. Kemper *et al*. [33] describe the specific methodology applied to Indo-Pacific bottlenose dolphins from SA. Two experienced readers each produced an age estimate which was then averaged.

### 2.7. Diet and biotoxin testing

The gastrointestinal contents of 26 Indo-Pacific bottlenose dolphins (14 males, 12 females) collected from SVG during 2013 were examined. These were processed using the methods described in Gibbs *et al*. [34]. Relative ages of the dolphins examined were neonate (*n* = 5), calf (*n* = 9), juvenile (*n* = 10), subadult (*n* = 1) and adult (*n* = 1).

Stomach contents of one Indo-Pacific bottlenose dolphin (M26206) were tested for 17 biotoxins (domoic acid, gymnodimine, azaspiracid-2, PTX, okadaic acid, DTX, yessotoxins, homoyessotoxins and brevetoxins) by the Cawthron Institute, Nelson, New Zealand.

### 2.8. Statistical methods

A goodness-of-fit *χ*^2^-test [35] was used to test the degree of emaciation in Indo-Pacific bottlenose dolphins during 2013 in SVG.

## 3. Results

### 3.1. Trends in strandings with evidence for the 2013 unusual mortality event

**Figure 1.**
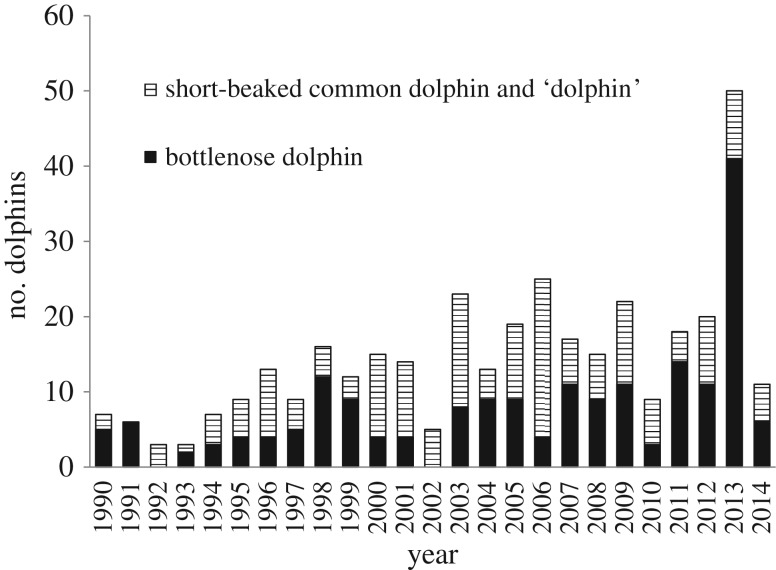
Annual counts of strandings for bottlenose dolphins, short-beaked common dolphins and unidentified dolphins ‘dolphin’ in SVG during 1990–2014 (*n* = 356).

Annual dolphin stranding records for 1990–2014 were compiled for SVG (figure 1). A substantial increase in reported strandings was evident in SVG during 2013, when 50 dolphins were recorded. Prior to 2013, SVG had a mean annual dolphin stranding count of 12.8 (range 3–25). For bottlenose dolphins, the mean annual count in SVG was 6.39 (0–14) and the 2013 count was 41. The 2014 strandings record returned to pre-2013 levels: 11 total dolphins, of which six were bottlenose dolphins. Annual count data elsewhere in SA (i.e. outside SVG) showed no peak in strandings during 2013: the 1990–2012 range was 9–41 (mean = 24.2) and in 2013 the count was 22.

**Figure 2.**
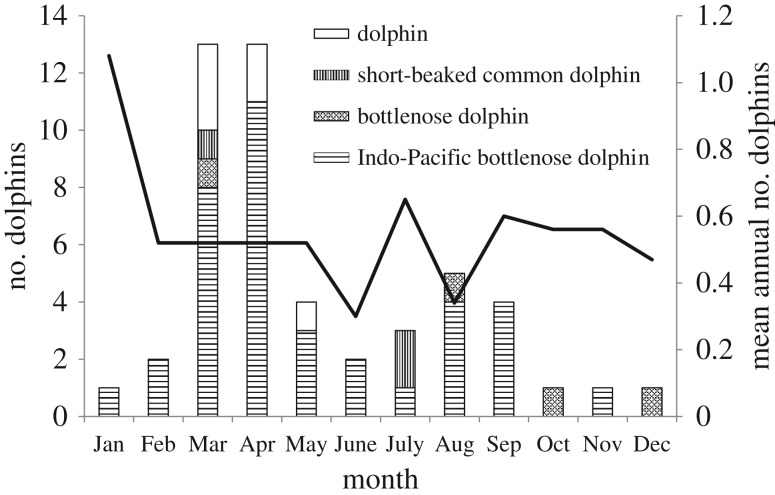
Monthly distribution of dolphin strandings in SVG. Histogram refers to 2013. Solid line denotes mean monthly strandings of bottlenose dolphins (*Tursiops* spp.) 1990–2012. Dolphin, unidentified dolphins.

**Figure 3.**
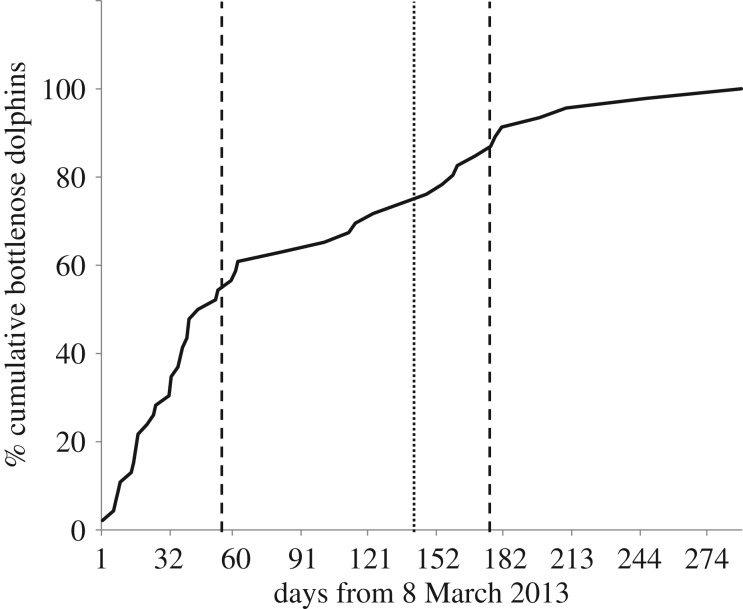
Cumulative increase (as percentage of total number of days of UME) in Indo-Pacific bottlenose dolphin strandings in SVG from 8 March 2013. Solid line, cumulative increase; dotted line, beginning of second peak; dashed line, end of both peaks.

The majority of SVG strandings during 2013 were bottlenose dolphins—all those collected were verified as the Indo-Pacific bottlenose dolphin. Short-beaked common dolphins were recorded in small numbers, as were unidentified dolphins (table 1). Three strandings (all Indo-Pacific bottlenose dolphins, only one collected) were reported in late January/early February. No strandings were reported from then until early March, when the UME commenced (figure 2). The initial, and larger, peak lasted until the end of the first week in May (approx. 60 days) during which time there was a steep rise in cumulative cases (figure 3). A second, smaller peak occurred during August and September, over a period of about 35 days. One mortality was recorded in each of October, November and December (all bottlenose dolphins), but these were not collected for examination.

### 3.2. Characterization of unusual mortality event

The preceding data provide evidence that a UME occurred in 2013 and that it primarily affected Indo-Pacific bottlenose dolphins in SVG. Here we summarize the demography and life history of the cases to gain insights into patterns within the UME. We also describe the stomach contents of the dolphins.

The seasonal pattern of strandings for bottlenose dolphins prior to the UME (1990–2012) showed the highest frequencies in January and a notable reduction from February to December (figure 2). During 2013 there was a different pattern, with a few strandings in January, followed by a substantial increase in March and April. During the latter months of 2013, there were fewer bottlenose strandings in SVG than at the same time in previous years. These findings provide additional evidence that 2013 was an atypical year for bottlenose dolphins in SVG.

Almost all the Indo-Pacific bottlenose dolphins that died during the early part of the UME were neonates, calves and juveniles (table 2). These had estimated ages of less than three months to 6 years (*n* = 20). The single subadult collected early in the UME had an estimated age of 6 years. Later, in the UME there was a more even spread of ages, with estimates of less than three months to 1 year for neonates and calves (*n* = 4) to 3–17 years for juveniles and adults (*n* = 6). Of the dolphins that died during the UME, 21 were males and 15 females. None of the collected females was pregnant or lactating.

**Table 2.** Relative age groups of Indo-Pacific bottlenose dolphins before and during the UME in SVG, SA. (See Material and methods for definition of age groups.)

	neonates	calves	juveniles	subadults	adults
pre-UME (Jan. 2012–Jan. 2013)	5	1	4	0	2
early UME (Mar.–early May 2013)	6	7	7	1	0
mid–late UME (late May–mid-Nov. 2013)	1	3	4	0	5

The seasonal pattern of relative age groups for the long-term dataset, which included both examined and not-examined carcasses, was compared between 1990–2012 and 2013. During the UME the ratio was skewed in favour of neonates, calves and juveniles for March to June 2013 (0–0.36) when compared with these monthly means prior to 2013 (0.41–0.71).

Of the 26 Indo-Pacific bottlenose dolphin gastrointestinal tracts examined, 15 stomachs contained food items. Fish remains included bones, teeth and eyeballs, a fin and a single otolith (Family Carangidae). Identifiable cephalopod remains (beaks) included octopus species (*n* = 74), cuttlefish (*n* = 19, *Sepia* sp.) and southern calamari, *Sepioteuthis australis* (*n* = 206). Very little soft tissue was present (3/8 stomachs i.e. remains of fish and/or cephalopods). The latter finding, plus the numerous stomachs with nil contents, indicates that many dolphins had not eaten just prior to death. Stomachs that contained milk were from neonates, calves and a single juvenile (body lengths of 115–169 cm).

### 3.3. Diagnostic investigations

A brief statement on the pathology findings for each dolphin is presented in the electronic supplementary material.

Of the 31 Indo-Pacific bottlenose dolphins from 2013 in SVG that could be assigned to a circumstance of death at post-mortem, most were categorized as infectious disease (*n* = 8) (see the electronic supplementary material). Follow-up histopathology showed that 23 had infectious disease as a major cause of death. None was related to anthropogenic causes. Many dolphins were found floating dead or suspected of having been floating dead before washing up because the skin on the extremities was flaccid and there was extensive skin damage by marine invertebrates.

The body condition of the 2013 Indo-Pacific bottlenose dolphins suggested both chronic and acute circumstances. Of the 23 cases where an index could be assigned, nine were robust and 14 were either slightly or severely emaciated. However, these findings were not statistically different from equality (*χ*^2 ^_1_= 1.09, *p* = 0.3). Of the 21 animals with infectious disease as a major cause of death, eight had a robust body condition, which suggests that the infectious condition was acute or subacute.

**Figure 4.**
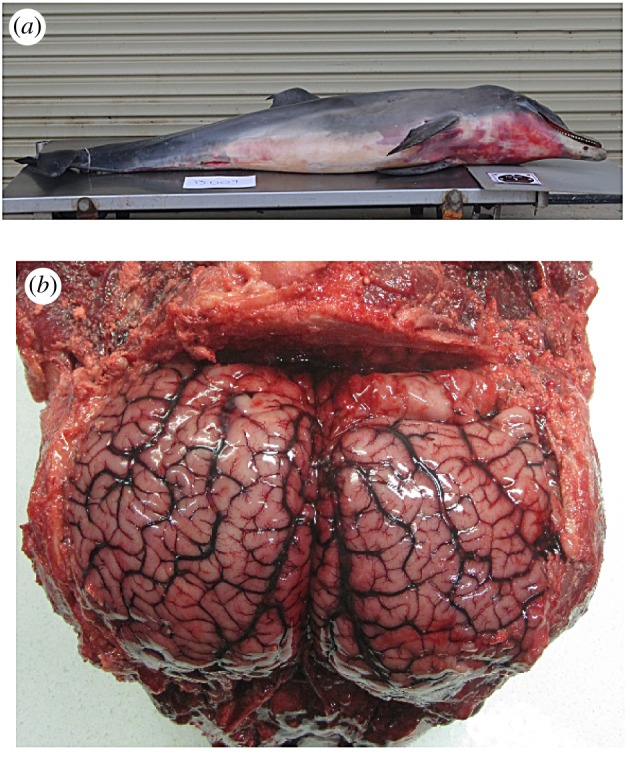
Images of some gross pathology findings in an Indo-Pacific bottlenose dolphin (M26204) collected during 2013 UME. (*a*) Severe cutaneous bruising in the head/neck region. This dolphin was very emaciated. (*b*) Brain showing blood vessel congestion on both hemispheres.

Severe or moderate cutaneous bruising was recorded in at least 65% of carcasses. In most cases, these lesions were found in the head/neck region (figure 4*a*) and were associated with subcutaneous tissue haemorrhage and oedema. Occasionally, bruising was noted in other parts of the body. Brains were examined from 13 dolphins and 11 showed local to extensive red discoloration of the meninges, sometimes with vascular distention (figure 4*b*) and/or oedema.

#### 3.3.1. Histopathology

**Figure 5.**
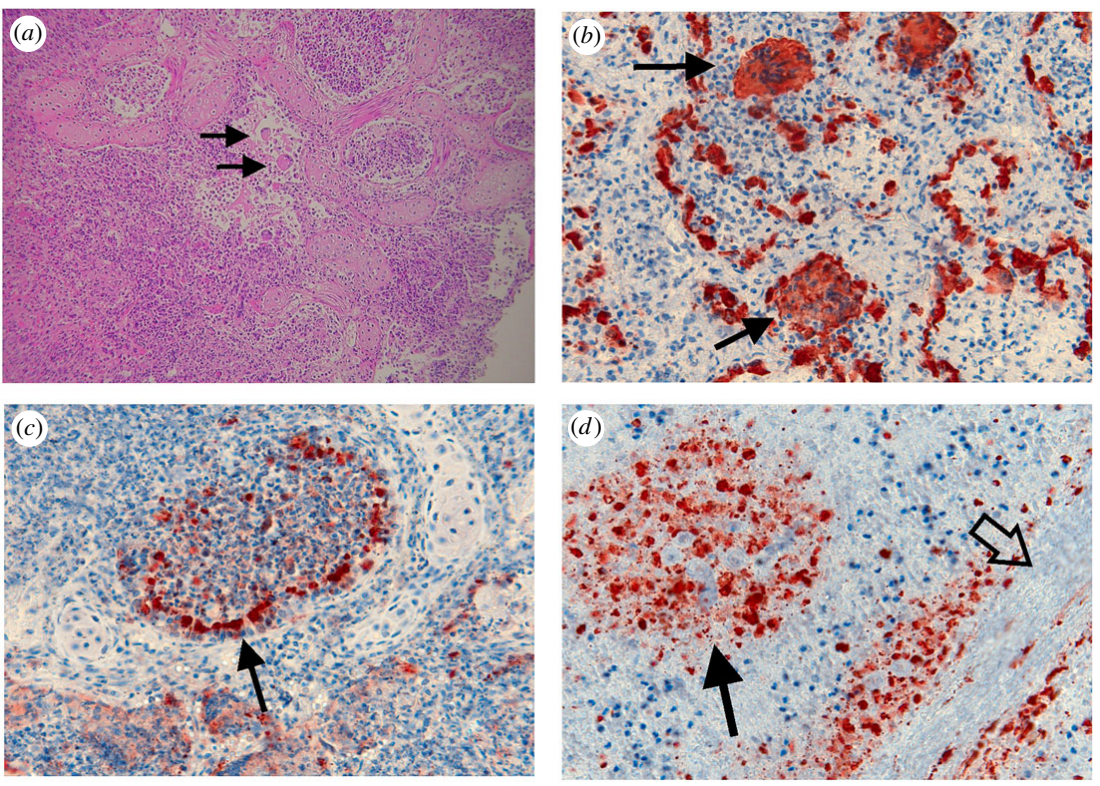
Photomicrographs of lung and spleen sample from Indo-Pacific bottlenose dolphins during the 2013 UME. (*a*) Lung (H&E) showing extensive inflammatory cell infiltration. Bronchial and alveolar spaces filled with inflammatory cells, exfoliated epithelial cells and pneumocytes. Syncytia (solid arrows) present. (*b*) Lung (IHC for morbillivirus) showing immunostaining (brown) for viral antigen present in syncytia (solid arrows) and alveolar epithelium. (*c*) Lung (IHC for morbillivirus) showing immunostaining for viral antigen (brown) in bronchiolar epithelium (solid arrow), and in epithelial and inflammatory cells in bronchial lumen and lung parenchyma. (*d*) Spleen (IHC for morbillivirus) showing diffuse staining for viral antigen (brown) in white pulp (solid arrow) alongside splenic trabeculum (open arrow).

Here we focus our examination on those mortalities involved in the UME, i.e. Indo-Pacific bottlenose dolphins that died in SVG during 2013. Moderate to severe interstitial bronchopneumonia was observed in 29 out of 30 (97%) of these dolphins. Lung lesions (figure 5*a*–*c*) generally had local to extensive inflammatory cell infiltration which resulted in disruption to the parenchyma, severe oedema/hyperplasia and extensive bronchial epithelial necrosis and sloughing, oedema associated with type 1 pneumocytes and hyperplasia of type 2 pneumocytes. Large syncytia were present in the lung parenchyma of 17 out of 30 (53%) dolphins (figure 5*a*,*b*). These lung lesions were generally (71%) associated with morbillivirus antigen (figure 5*a*–*c*). Lymphoid depletion occurred in 18 out of 24 (75%) of animals for which this tissue was available. Of the 13 brains available, four showed local to extensive suppurative, necrotizing meningioencephalitis. These pathologies are often associated with morbillivirus.

Within these cases, lesions in various other tissues were observed, including hepatic abscessation, pericarditis, myocarditis, nephrosis and ulceration of the stomach. Lesions were also observed in association with parasites, including parasitic hepatitis (trematodes), protozoal encephalitis (*Toxoplasma gondii*) and pneumonia (lung nematodes). Lung nematodes were also confirmed by whole animals extracted from tissue. In all, these lesions, which are not directly attributable to morbillivirus, were observed in 25 out of 30 (83%) Indo-Pacific bottlenose dolphins collected from SVG during 2013.

Histopathology for other dolphin cases (all species from outside SVG (*n* = 14) and from SVG prior to 2013 (*n* = 13)) showed limited similarity to that of the UME dolphins. Within this subsample, two short-beaked common dolphins had severe interstitial bronchopneumonia and lymphoid depletion (see the electronic supplementary material). The morbillivirus positive (IHC and PCR) common bottlenose dolphin from the southeast of SA also showed similar lesions. Large syncytia were not found in any of the non-UME dolphins. Prior to the UME, brains were not studied and samples were not collected for bacterial and fungal culture.

#### 3.3.2. Morbillivirus and other pathogen testing

Of 57 dolphin carcasses recorded from around SA between 2005 and 2013, and tested for morbillivirus by IHC and/or RT-PCR, 46 were positive by either or both tests (table 3). Of the 46 positives, 20 were confirmed in both tests and 26 were positive by PCR only (including one not tested by IHC). No dolphin was positive on IHC and negative on PCR. Of the 29 Indo-Pacific bottlenose dolphins collected and tested during the period and geographical boundaries of the UME, all were positive by PCR and 19 positive by both tests.

**Table 3.** Morbillivirus PCR and IHC results for three dolphin species in SA collected during 2005–2013. (Numbers to the left of the forward slash are positive dolphins; numbers to the right are dolphins tested.)

	2005–2010		2011–2012		2013		total	
species	PCR	IHC	PCR	IHC	PCR	IHC	PCR	IHC
Indo-Pacific bottlenose dolphin								
SVG	0/1	0/2	4/6	0/4	29/30^a^	19/28	33/37	19/34
outside SVG	0/1	0/1	0/0	0/0	2/4	0/4	2/5	0/5
common bottlenose dolphin	0/0	0/0	0/0	0/0	1/2	1/1	1/2	1/1
short-beaked common dolphin	0/2	0/2	2/2	0/2	8/8	0/8	10/12	0/12

^a^Negative dolphin collected in January 2013, prior to the beginning of UME.

In lungs, viral antigen was located locally to diffusely in bronchial and alveolar epithelium, in cellular material sloughed into the bronchial lumen or alveoli, in syncytia, in fibrous tissue, in smooth muscle bundles and in single cells within inflammatory tissue (figure 5*b*,*c*). Antigen was associated with inflammatory changes in lung parenchyma and airways. It was prominent in fibrous tissue surrounding chronic inflammatory lesions associated with abscesses and parasites. In a few cases where other tissues were examined, antigen was also found in the splenic white pulp and trabeculae (figure 5*d*); in the submucosa, lamina propria, crypt epithelium and lymphatic nodules of the intestine; in perivascular connective tissue at various sites and in isolated neurons in the brain.

Of 56 dolphins tested by PCR, 48 were positive. Amplified PCR products, approximately 425 bp of phosphoprotein (P) gene, were sequenced and compared with sequences of known CeMV and other morbilliviruses. Sequence analysis demonstrated 99.7% and 79.2% nucleotide identity to the CeMV from Indo-Pacific bottlenose dolphins from Western Australia [10], and to the CeMV from a common bottlenose dolphin from Queensland [15], respectively.

Bacteria that are known to be pathogenic were identified in three Indo-Pacific bottlenose dolphins (each dolphin with a single type): samples from a skin lesion (*Pasteurella* spp.), blowhole (*Salmonella* spp.) and liver (*Streptococcus* spp.). A pathogenic fungus (*Aspergillus fumigatus*) was identified in the lung of two dolphins.

#### 3.3.3. Toxicology

The stomach contents of one juvenile Indo-Pacific bottlenose dolphin (collected 1 April 2013 in SVG) were analysed for 17 toxins (see Material and methods for list of toxins) that can be produced during harmful algal blooms. Results were negative at detection limits of less than 0.01–0.05 mg kg^−1^.

### 3.4. Epidemiology of dolphin morbillivirus

**Figure 6.**
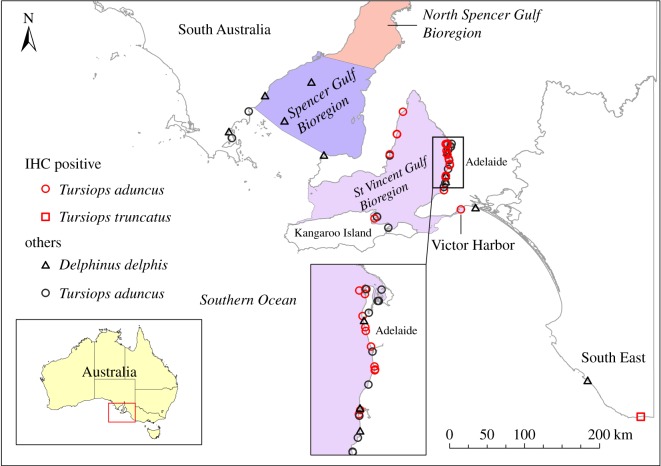
Geographical distribution of dolphin carcasses testing positive for morbillivirus in SA during 2005–2013. Red symbols, IHC-positive; black symbols, PCR positive and IHC negative or not tested.

Molecular evidence for morbillivirus was found in dolphins collected from wide-ranging locations in SA, including Spencer Gulf, SVG and southeastern SA (figure 6). However, only one dolphin was tested from far western SA and it was negative for CeMV. IHC-positive dolphins were from SVG and were recorded throughout that bioregion. The only other IHC-positive carcass was from the southeast of SA.

**Figure 7.**
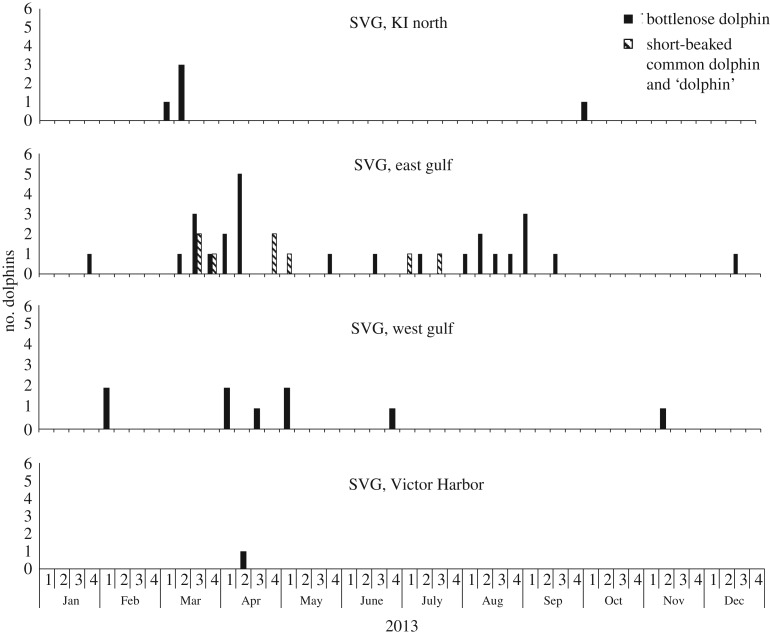
Geographical pattern of strandings in 2013 UME in SVG. Solid bars, Indo-Pacific bottlenose dolphin; hatched bars, short-beaked common dolphin and unidentified dolphins ‘dolphin’; KI, Kangaroo Island.

An analysis of the geographical pattern of strandings during 2013 (figure 7) may demonstrate how a pathogen moved through the population. The first mortality was reported on 8 March on the north coast of Kangaroo Island (KI). A week later carcasses were reported on the eastern coast of SVG, primarily in the Adelaide region. From 8 April, mortalities began to appear on the western side of SVG. Mortalities on the north coast of KI that showed evidence of morbillivirus were reported only during the first half of March, which suggests that the virus did not persist there. Eastern SVG was the primary area affected by the virus.

Morbillivirus was identified only in carcasses collected during 2011–2013 and during this time, 90% (combining three species) were PCR positive for CeMV (table 3). The first IHC-positive dolphin was collected on 8 March 2013 and the last on 1 September 2013. No dolphins were studied that were collected after 2013.

PCR evidence for CeMV was found in 35 out of 42 (83%) of Indo-Pacific bottlenose dolphins from SA during 2011–2013, with the first cases appearing in 2011 and 2012 in SVG but not elsewhere. Fewer dolphins were IHC-positive (19 out of 39, 49%) for the virus (table 3) and IHC positive dolphins were collected only during 2013. Of the two common bottlenose dolphins studied, only one had evidence of morbillivirus (both PCR and IHC). Evidence for morbillivirus (PCR only) was found in a large proportion of short-beaked common dolphins (10 out of 12, 83%) studied. The virus was not detected until 2011–2012, although only two short-beaked common dolphins were studied prior to this.

During the UME all the Indo-Pacific bottlenose dolphin calves and juveniles were PCR positive for CeMV (electronic supplementary material). Two dolphins that were considered less than a day old were PCR positive for CeMV, which is evidence for vertical transmission of the virus. For non-UME dolphins (all species, all regions) there was a bias in favour of young animals prior to 2013 (*n* = 13). During 2013, the ages were more evenly distributed (*n* = 14). Of the two common bottlenose dolphins studied, the adult was positive (PCR and IHC) and the neonate was PCR CeMV negative.

## 4. Discussion

In this study, we demonstrate that the 2013 dolphin UME in SA was associated with morbillivirus infection. Although other infectious conditions were present in affected animals, the UME clearly coincided with the appearance of severe morbillivirus bronchopneumonia, and we conclude that morbillivirus was a major causative agent for the event. This UME is, to our knowledge, the first large marine mammal event linked to morbillivirus in the Southern Hemisphere. One other CeMV-related UME has been documented in Australia and another is suspected. Both were in Western Australia and involved Indo-Pacific bottlenose dolphins from small resident populations. The Swan River case [9,10] documented six mortalities from a population of 20-25 dolphins and this exceeded the annual mean by six-fold. As in the SA UME, the Swan River 2009 event had two temporal clusters of mortalities but these involved different causes of death—the first related to CeMV and the second to extreme ulcerative dermatitis associated with presumed poxvirus infection [9,10]. A suspected UME occurred in Indo-Pacific bottlenose dolphins near Bunbury, Western Australia, during a 13-month period in 2008–2009. Four confirmed and three presumed mortalities were recorded out of 14 resident dolphins [12]. Post-mortem examinations on four of the dolphins showed pathological evidence (lymphoid depletion, secondary bacterial and fungal infections, lungworm infections, poor body condition) similar to the Swan River UME, which suggests that the event was linked to CeMV.

Until the discovery of the Australian cases, bottlenose dolphins were involved in UMEs only along the east coast of the USA [2]. Of the 18 cases in the USA since 1991, the cause of over 70% was considered undetermined, about 20% were caused by biotoxins and one was believed to be related to infectious disease. However, some of the events listed as ‘undetermined’ were, at least in part, linked to morbillivirus: five confirmed and two suspected [13,36,37].

In a recent review of morbillivirus in cetaceans, Van Bressem *et al*. [3] summarized its distribution worldwide, including some of the results of the present study. In the Southern Hemisphere, the virus has been recorded in the Indian, South Pacific (eastern and western), South Atlantic and Southern Oceans, but only in Brazil and Australia has the antigen been identified [3,38]. In Australia, antibodies to morbillivirus have been identified in common bottlenose dolphins collected from Tasmania in 1997 [7] and in five cetacean species, including the common bottlenose dolphin and Indo-Pacific bottlenose dolphin, from Queensland and northern New South Wales post-1984 [14]. Pathological evidence for the disease, with detection of the virus using IHC, was first reported in Indo-Pacific bottlenose dolphins in Western Australia in 2009 [9,10], in a single common bottlenose dolphin from Queensland in 2010 [15], and in both these species during 2013 in this study.

The distribution of morbillivirus in infected dolphins collected from SVG during 2013 would have contributed significantly to morbidity in most cases. In dolphins that were PCR positive but IHC negative, the infection may have been limited to body regions that were not sampled for IHC, they may have been mild infections with low antigen loads or they may have been convalescing following non-fatal infection. Severe cutaneous bruising was frequently observed in dolphins that died during the 2013 UME and may also have contributed to mortality; however, its cause was not ascertained. This condition has not been described in other studies of morbillivirus-related UMEs.

There does not appear to be a consistent pattern to UMEs involving infectious disease: they are variable in geographical extent, number of mortalities and duration [3]. Rubio-Guerri *et al*. [39] compared the pattern of cumulative mortality of dolphins (mostly striped dolphins, *Stenella coeruleoabla*) in the Mediterranean Sea during three UMEs separated by 11 years. The first two were characterized by a slow response [40]. The final UME, in 2011, involved slightly fewer dolphins (37 versus approx. 50) and had a rapid increase in mortalities at the beginning of the event, similar to the SA 2013 UME. Fauquier *et al*. [36] documented an extensive UME (mostly of common bottlenose dolphins) along the east coast of the USA during 2013 and 2014. It involved over 1250 dolphins and lasted at least 10 months. The pattern of mortalities was similar to the SA event in terms of an initial rapid increase but no second peak was observed during the USA event.

Unlike UMEs caused by biotoxins, those linked to infectious disease are likely to be complex and vary according to environmental and host factors. Environmental variables (e.g. fisheries interactions, toxic contaminants, higher than normal sea surface temperatures, limited prey availability) play a role [13]. Host population structure, size and dynamics (e.g. inbreeding, social structure, migration) and contact with other species are likely to also be important factors [13,37,41]. The Indo-Pacific bottlenose dolphin is a resident, inshore species forming loose associations that occupy home ranges of up to 200 km along coasts with suitable habitat [42,43]. There is potential, therefore, for a rapid spread of infectious disease and this appeared to be the case in SVG during 2013. Preliminary evidence shows that some Indo-Pacific bottlenose dolphins make movements of at least 100 km in SVG (T. Bartram 2015, personal communication). Limited gene flow between SVG and other parts of SA [44] may, in part, explain why the UME was confined to that region. However, since CeMV was identified by PCR in SVG and elsewhere in SA before 2013, some factor, or factors, must have triggered the UME. There are no population estimates for Indo-Pacific bottlenose dolphins in SVG to indicate that population density was higher than normal before or during 2013.

The dolphin UME coincided with a major climatic anomaly in south eastern Australia, including SA, that resulted in water temperatures substantially higher (3–5°C) than normal [45]. Resulting phytoplankton blooms were monitored sporadically in SVG, with the primary species being a barbed diatom, *Chaetoceros coarctatus*. During late February to early April 2013, there were at least 17 fish die-offs in SA, mostly SVG. The fish died as a result of bacterial infection resulting from gill damage caused by this diatom [45]. There was no evidence that the fish or the dolphins died as result of biotoxins produced during a harmful algal bloom. Although these have resulted in the deaths of large numbers of bottlenose dolphins elsewhere [46–48], there are no documented cases of this agent killing marine mammals in Australia.

The fish involved in the die-offs were primarily small benthic species [45], none of which was identified in the stomachs of the Indo-Pacific bottlenose dolphins collected during the 2013 UME. One of the contributing factors to the UME may have been reduced prey availability, although this is speculative. Cephalopod beaks were the most numerous items in the examined stomachs. These are resistant to digestion and can remain in the stomach of a marine mammal for longer than otoliths [49]. The lack of undigested prey indicated the dolphins had not consumed food just prior to death, which is additional evidence that an agent resulting in acute mortality (e.g. a biotoxin) was not involved in the UME.

Toxic contaminants are sometimes included in the possible factors contributing to UMEs of marine mammals [41]. Organochlorines and other lipid-soluble toxins may act through immune compromise [50]. There has been limited analysis of these compounds in SA dolphins with a few elevated concentrations reported, e.g. PCB Arochlor 1260 up to 19.5 mg kg^−1^ wet weight in an Indo-Pacific bottlenose dolphin from SVG [51]. Much more is known about the concentrations of heavy metals in SA dolphins. Lavery *et al*. [52] showed that levels of zinc, cadmium, mercury and lead are found in high concentrations in some Indo-Pacific bottlenose dolphins from the SA gulfs. An interesting future study would be to determine the concentrations of heavy metals in the Indo-Pacific bottlenose dolphins that died during the 2013 UME.

A large proportion of young (82%, less than 7 years) compared with older (18%, 12–17 years) Indo-Pacific bottlenose dolphins died during the UME in 2013, which is in contrast with some other studies. Chabanne *et al*. [9] noted mixed age groups in the first peak of the Western Australian UME and only adults in the second. A very large UME of common bottlenose dolphins in the USA included all age groups [36]. Epizootics of *S. coeruleoalba* in the Mediterranean Sea have involved various combinations of age groups [39,40,53]. Biotoxin-related UMEs involve a relatively even distribution of age classes for marine mammals [6,48].

Domingo *et al*. [5] discussed two scenarios relating to morbillivirus infections in marine mammals: (i) severe and with high mortality, the virus subsequently disappearing from the population and (ii) more or less severe with susceptible hosts always available for infection and epizootics cease because most individuals become immune from an early age. The disease can then become endemic in scenario two and would involve infected individuals over a considerable period of time [5]. Future testing of archived samples (SAM), collected since 1985 from many cetacean species, would help to describe the prevalence and nature of disease processes of morbillivirus, brucellosis and toxoplasmosis in SA marine mammals.

Why has it taken so long for a major mortality event to occur in Australia when morbillivirus was identified in cetaceans collected as early as 1985 [14]? Van Bressem *et al*. [13] noted that pollution may increase the frequency of disease in marine mammals. This observation may explain why the North Atlantic has experienced so many large marine mammal UMEs since the environment there has been polluted by human activities for hundreds of years. By contrast, the Australian marine environment is generally unpolluted and pinnipeds and cetaceans inhabiting these waters may be more resilient to epizootics. Recurring UMEs associated with morbillivirus are known in many parts of the world [13,40] and are likely to occur in Australian cetaceans in the future.

## Supplementary Material

Electronic Supplementary Data: Details of collected carcasses examined in this study from SA during 2005–2013. NE = not examined. SVG-E = SVG east, SVG-W = SVG west, SVG-KI = SVG Kangaroo Island, SVG-VH = SVG Victor Harbor, SE = far south east of SA, SG = Spencer Gulf, WSA = western SA. Circumstance of death is assigned at post-mortem. Major cause of death is based on gross and histopathology. NA= not available, NE = not examined, MV = morbillivirus. The short description is as follows: Details of collected carcasses examined in this study.

## Data accessibility

The dataset supporting this article has been uploaded as the electronic supplementary material.
