# Supplementary material for: Morbillivirus-associated unusual mortality event in South Australian bottlenose dolphins is largest reported for the Southern Hemisphere
Source: R Soc Open Sci. 2016 Dec 21;3(12):160838. doi: 10.1098/rsos.160838 (PMC5210697; doi:10.1098/rsos.160838)
Supplement: Electronic Supplementary Data: Details of collected carcasses examined in this study from SA during 2005–2013. NE = not examined. SVG-E = SVG east, SVG-W = SVG west, SVG-KI = SVG Kangaroo Island, SVG-VH = SVG Victor Harbor, SE = far south east of SA, SG = Spencer Gulf, WSA = western SA. Circumstance [file rsos160838supp1.docx]

Electronic Supplementary Data: Details of collected carcasses examined in this study from SA during 2005–2013. NE = not examined. SVG-E = SVG east, SVG-W = SVG west, SVG-KI = SVG Kangaroo Island, SVG-VH = SVG Victor Harbor, SE = far south east of SA, SG = Spencer Gulf, WSA = western SA. Circumstance of death is assigned at post-mortem. Major cause of death is based on gross and histopathology. NA= not available, NE = not examined, MV = morbillivirus.

| species | date | location | specimen no. | sex | total length (cm) | age | circumstance death | major cause death | pneumonia | lymphoid depletion | bacterial, fungal, parasitic infection | MV RT-PCR | MV IHC |
| --- | --- | --- | --- | --- | --- | --- | --- | --- | --- | --- | --- | --- | --- |
| short-beaked common dolphin | 28/5/2005 | SVG-E | M23911 | M | 119 | Calf | Unknown (possible cutaneous bruising) | traumatic injury | yes | NE | present | neg | neg |
| short-beaked common dolphin | 13/5**/**2006 | SVG-W | M24346 | M | 108 | Calf | Unknown (cutaneous bruising, moderate lung nematode infection) | parasitic pneumonia | yes | absent | present | neg | neg |
| short-beaked common dolphin | 17/1/2012 | Coorong | 12.045 | M | 109 | Juvenile | Unknown (cutaneous bruising) | traumatic injury | yes | NE | present | pos | neg |
| short-beaked common dolphin | 28/10/2012 | SG | 13.042 | M | 115 | Calf | Unknown (possible entanglement, possible chronic disease) | traumatic injury, emaciation | yes | present | absent | pos | neg |
| short-beaked common dolphin | 28/3/2013 | SVG-E | 13.018 | M | ~100 | Neonate (<1 day) | Infectious Disease (severe lung nematode infection) | parasitic pneumonia | yes | absent | present | pos | neg |
| short-beaked common dolphin | 14/4/2013 | SG | 13.040 | M | 148 | Juvenile | Known Entanglement (SA Sardine Fishery) | drowning | yes | absent | absent | pos | neg |
| short-beaked common dolphin | 17/4/2013 | SE | 13.033 | F | 129 | Juvenile | Non-infectious Disease (live stranded, icterus) | icterus | yes | absent | absent | pos | neg |
| short-beaked common dolphin | 19/4/2013 | SG | 13.041 | F | 182 | Subadult | Probable Entanglement | drowning | no | absent | present | pos | neg |
| short-beaked common dolphin | 19/5/2013 | SG | 13.044 | F | 198 | Subadult | Unknown (possible entanglement, acute death) | suffocation | no | present | present | pos | neg |
| short-beaked common dolphin | 20/5/2013 | SG | 13.048 | M | 164 | Juvenile | Known Entanglement (SA Sardine Fishery) | suffocation, circulatory disorder | yes | absent | present | pos | neg |
| short-beaked common dolphin | 2/7/2013 | SVG-E | 13.055 | F | 195 | Adult | Probable Entanglement | traumatic injury | yes | present | present | pos | neg |
| short-beaked common dolphin | 22/7/2013 | SVG-E | 13.056 | M | 95 | Neonate | Disease (icterus, severe lung nematode infection) | icterus | yes | NE | present | pos | neg |
| Indo-Pacific bottlenose dolphin | 30/11/2005 | WSA | M24285 | F | 139 | Calf | Known Entanglement (shark net) | suffocation, circulatory disorder | yes | NE | absent | neg | neg |
| Indo-Pacific bottlenose dolphin | 23/11/2009 | SVG-E | M24899 | F | 150 | Juvenile | Non-infectious Disease (chronic kidney disease, severe cutaneous bruising | generalised infection | yes | absent | present | NE | neg |
| Indo-Pacific bottlenose dolphin | 14/5/2010 | SVG-E | M25054 | M | 190 | Juvenile | Intentional Killing (shotgun pellet) | pleuritis | yes | absent | present | neg | neg |
| Indo-Pacific bottlenose dolphin | 25/2/2011 | SVG-E | 11.015 | M | 104 | Neonate (<3 months) | Unknown (severe cutaneous bruising, alive after birth) | traumatic injury | yes | absent | absent | pos | neg |
| Indo-Pacific bottlenose dolphin | 9/8/2011 | SVG-E | M26266 | F | 122 | Calf | Non-infectious Disease (emaciated, multi-organ disease) | emaciation | yes | absent | present | pos | neg |
| Indo-Pacific bottlenose dolphin | 1/9/2011 | SVG-KI | 12.037 | M | 189 | Juvenile | Unknown (cutaneous bruising, icterus) | icterus | no | absent | absent | neg | NE |
| Indo-Pacific bottlenose dolphin | 24/2/2012 | SVG-E | 12.050 | M | 106 | Neonate | Unknown (penetrating trauma, icterus) | icterus | yes | NE | present | pos | neg |
| Indo-Pacific bottlenose dolphin | 9/3/2012 | SVG-KI | M26197 | M | 95 | Neonate (<3 months) | Unknown (moderate cutaneous bruising, possible disease) | emaciation | yes | absent | absent | pos | neg |
| Indo-Pacific bottlenose dolphin | 27/7/2012 | SVG-E | 12.085 | M | 126 | Juvenile | Unknown (chronic disease, trauma) | suffocation | yes | NE | present | pos | neg |
| Indo-Pacific bottlenose dolphin | 29/1/2013 | SVG-E | M26205 | F | 186 | Juvenile (3 years) | Unknown (cutaneous bruising, possible entanglement?) | generalised infection | NE | present | present | neg | NE |
| Indo-Pacific bottlenose dolphin | 4/2/2013 | SG | M26192 | M | 232 | Subadult (13 years) | Known Entanglement (Kingfish Aquaculture Farm) | drowning | yes | absent | absent | pos | neg |
| Indo-Pacific bottlenose dolphin | 15/2/2013 | SG | M26193 | M | 225 | Adult (>12 years) | Disease (live stranded, abnormal testes, pathogenic bacteria in blowhole, multi-organ disorder) | generalised infection | yes | NE | present | neg | neg |
| Indo-Pacific bottlenose dolphin | 15/2/2013 | SG | M26194 | M | 224 | Adult (>12 years) | Infectious Disease (live tranded, abdominal infection) | generalised infection | yes | NE | present | neg | neg |
| Indo-Pacific bottlenose dolphin | 5/3/2013 | SG | M26195 | M | 100 | Neonate (<3 months) | Unknown (neonatal, alive after birth, possible entanglement) | meconium aspiration syndrome/ birth stress | no | present | absent | pos | neg |
| Indo-Pacific bottlenose dolphin | 8/3/2013 | SVG-KI | M26011 | F | 153 | Juvenile (1 year) | Disease (severe cutaneous bruising, multi-organ disease, skin lesions) | pneumonia, emaciation | yes | NE | present | pos | pos |
| Indo-Pacific bottlenose dolphin | 14/3/2013 | SVG-E | M26204 | F | 165 | Juvenile (3 years) | Infectious Disease (chronic condition, lung nematode infection) | pneumonia | yes | present | present | pos | pos |
| Indo-Pacific bottlenose dolphin | 15/3/2013 | SVG-KI | M26201 | M | 96 | Neonate (< 3 months, possible still birth) | Unknown (neonatal, severe cutaneous bruising) | birth stress | yes | NE | absent | pos | neg |
| Indo-Pacific bottlenose dolphin | 16/3/2013 | SVG-KI | M26198 | F | 93 | Neonate (<3 months, probable still birth) | Unknown (neonatal, severe cutaneous bruising) | pneumonia | yes | absent | absent | pos | neg |
| Indo-Pacific bottlenose dolphin | 24/3/2013 | SVG-E | M26190 | F | 154 | Juvenile (1 year) | Infectious Disease (heart and lung infection, severe cutaneous bruising) | pneumonia | yes | NE | present | pos | pos |
| Indo-Pacific bottlenose dolphin | 24/3/2013 | SVG-E | M26196 | M | 115 | Neonate (~3 months) | Disease (severe cutaneous bruising, lung nematode infection) | parasitic pneumonia, emaciation | yes | NE | present | pos | pos |
| Indo-Pacific bottlenose dolphin | 24/3/2013 | SVG-E | M26006 | F | 128 | Calf (3 months–1 year) | Unknown (severe cutaneous bruising) | pneumonia | yes | present | present | pos | pos |
| Indo-Pacific bottlenose dolphin | 31/3/2013 | SVG-E | M26202 | M | 121 | Calf (~3 months–1 year) | Unknown (severe cutaneous bruising, moderate lung nematode infection) | pneumonia | yes | present | present | pos | pos |
| Indo-Pacific bottlenose dolphin | 1/4/2013 | SVG-E | M26206 | F | 169 | Juvenile (3 years) | Unknown (severe cutaneous bruising, chronic lung nematode infection, possible other disease) | pneumonia | yes | absent | present | pos | pos |
| Indo-Pacific bottlenose dolphin | 7/4/2013 | SVG-W | M26001 | M | 174 | Juvenile (6 years) | Infectious Disease (severe cutaneous bruising, multi-organ disease) | pneumonia | yes | present | present | pos | pos |
| Indo-Pacific bottlenose dolphin | 8/4/2013 | SVG-W | M26189 | F | 169 | Juvenile (1 year) | Unknown (severe congestion to brain, possible infection) | parasitic encephalitis, emaciation | yes | present | present | pos | neg |
| Indo-Pacific bottlenose dolphin | 8/4/2013 | SVG-E | M26191 | F | 207 | Subadult (6 years) | Unknown (severe cutaneous bruising) | traumatic injury | yes | absent | present | pos | neg |
| Indo-Pacific bottlenose dolphin | 11/4/2013 | SVG-E | M26188 | M | 148 | Calf (1 year) | Unknown (possible disease, moderate cutaneous bruising) | pneumonia | yes | present | present | pos | pos |
| Indo-Pacific bottlenose dolphin | 13/4/2013 | SVG-E | M26012 | M | 104 | Neonate (2–3 months) | Unknown (penetrating and cutaneous bruising) | traumatic injury | yes | absent | absent | pos | neg |
| Indo-Pacific bottlenose dolphin | 13/4/2013 | SVG-E | M26013 | M | 102 | Neonate (~5 weeks) | Infectious Disease (large lung abscess, pleural infection) | generalised infection | yes | absent | present | pos | neg |
| Indo-Pacific bottlenose dolphin | 15/4/2013 | SVG-E | M26199 | F | 135 | Calf (3 months–1 year) | Infectious Disease (heart infection, moderate lung nematode infection, cutaneous bruising) | generalised infection | yes | present | present | pos | pos |
| Indo-Pacific bottlenose dolphin | 16/4/2013 | SVG-VH | M26014 | F | 146 | Calf (3 months–1 year) | Unknown (possible chronic disease, moderate lung nematode infection, severe cutaneous bruising) | pneumonia | yes | NE | present | pos | pos |
| Indo-Pacific bottlenose dolphin | 16/4/2013 | SVG-E | M26015 | M | 121 | Neonate (<3 months) | Unknown (mild lung nematode infection, severe cutaneous bruising, possible other disease) | pneumonia | yes | present | present | pos | pos |
| Indo-Pacific bottlenose dolphin | 20/4/2013 | SVG-W | M26200 | M | 115 | Calf (3 months–1 year) | Unknown (severe cutaneous bruising, lung nematode infection) | emaciation | yes | NE | present | pos | pos |
| Indo-Pacific bottlenose dolphin | 7/5/2013 | SVG-W | M26016 | F | 127 | Calf (3 months–1 year) | Unknown (deep cutaneous bruising, possible disease) | pneumonia | yes | absent | absent | pos | pos |
| Indo-Pacific bottlenose dolphin | 28/5/2013 | SVG-E | M26017 | F | 130 | Calf (3 months–1 year) | Unknown (moderate lung nematode infection, possible trauma, possible disease) | emaciation | yes | present | present | pos | NE |
| Indo-Pacific bottlenose dolphin | 19/6/2013 | SVG-E | M26004 | F | 160 | Juvenile (3 years) | Unknown (chronic lung nematode infection, severe cutaneous bruising) | emaciation, pneumonia | yes | present | present | pos | pos |
| Indo-Pacific bottlenose dolphin | 30/6/2013 | SVG-W | M26008 | M | 138 | Calf (3 months–1 year) | Unknown (chronic lung nematode infection, mild cutaneous bruising) | pneumonia, lymphadenitis | yes | present | present | pos | pos |
| Indo-Pacific bottlenose dolphin | 3/8/2013 | SVG-E | M26005 | M | 195 | Juvenile (7 years) | Unknown (severe cutaneous bruising, possible disease) | traumatic injury | yes | present | present | pos | pos |
| Indo-Pacific bottlenose dolphin | 15/8/2013 | SVG-E | M26007 | M | 225 | Adult (17 years) | Disease (multi-organ, icterus) | icterus, generalised infection | yes | present | present | pos | neg |
| Indo-Pacific bottlenose dolphin | 17/8/2013 | SVG-E | M26003 | M | NA | Adult (16 years) | Infectious Disease (live stranded, lung nematode infection) | pneumonia, encephalitis | yes | present | present | pos | pos |
| Indo-Pacific bottlenose dolphin | 25/8/2013 | SVG-E | M26002 | M | 208 | Juvenile (12 years) | Infectious Disease (cutaneous bruising, multi-organ disease) | generalised infection | yes | present | present | pos | neg |
| Indo-Pacific bottlenose dolphin | 1/9/2013 | SVG-E | M26009 | M | 141 | Calf (3 months–1 year) | Infectious Disease (chronic multi-organ disease, shot gun pellet, severe cutaneous bruising) | parasitic pneumonia, lymphoid depletion | yes | present | present | pos | pos |
| Indo-Pacific bottlenose dolphin | 6/9/2013 | SVG-E | M26203 | M | 213 | Juvenile (15 years) | Unknown (acute death, severe cutaneous bruising) | traumatic injury | yes | present | present | pos | neg |
| Indo-Pacific bottlenose dolphin | 23/9/2013 | SVG-E | M26010 | M | >52 | Neonate (<3 months) | Unknown (neonatal) | organs not available | NE | NE | NE | NE | NE |
| common bottlenose dolphin | 28/1/2013 | SE | M26207 | M | 129 | Neonate (<4 months) | Unknown (advanced decomposed) | unknown | NE | NE | absent | neg | NE |
| common bottlenose dolphin | 20/3/2013 | SE | M26000 | F | 267 | Adult (~20–25 years) | Infectious Disease (chronic lung nematode infection) | generalised infection | yes | present | present | pos | pos |
